# Characterizing genetic variation in the regulation of the ER stress response through computational and *cis*-eQTL analyses

**DOI:** 10.1093/g3journal/jkad229

**Published:** 2023-10-04

**Authors:** Nikki D Russell, Lynn B Jorde, Clement Y Chow

**Affiliations:** Department of Human Genetics, University of Utah School of Medicine, Salt Lake City, UT 84112, USA; Department of Human Genetics, University of Utah School of Medicine, Salt Lake City, UT 84112, USA; Department of Human Genetics, University of Utah School of Medicine, Salt Lake City, UT 84112, USA

**Keywords:** ER stress, genetic variation, *cis*-eQTL, transcriptional variability, genetic resource

## Abstract

Misfolded proteins in the endoplasmic reticulum (ER) elicit the ER stress response, a large transcriptional response driven by 3 well-characterized transcription factors (TFs). This transcriptional response is variable across different genetic backgrounds. One mechanism in which genetic variation can lead to transcriptional variability in the ER stress response is through altered binding and activity of the 3 main TFs: XBP1, ATF6, and ATF4. This work attempts to better understand this mechanism by first creating a computational pipeline to identify potential binding sites throughout the human genome. We utilized GTEx data sets to identify *cis*-eQTLs that fall within predicted TF binding sites (TFBSs). We also utilized the ClinVar database to compare the number of pathogenic vs benign variants at different positions of the binding motifs. Finally, we performed a *cis*-eQTL analysis on human cell lines experiencing ER stress to identify *cis*-eQTLs that regulate the variable ER stress response. The majority of these *cis*-eQTLs are unique to a given condition: control or ER stress. Some of these stress-specific *cis*-eQTLs fall within putative binding sites of the 3 main ER stress response TFs, providing a potential mechanism by which these *cis*-eQTLs might be impacting gene expression under ER stress conditions through altered TF binding. This study represents the first *cis*-eQTL analysis on human samples experiencing ER stress and is a vital step toward identifying the genetic components responsible for the variable ER stress response.

## Introduction

The endoplasmic reticulum (ER) is a large eukaryotic organelle and is a major site of protein and lipid synthesis, protein folding, and calcium storage (Alberts et al. 2002; Schwarz and Blower 2016). ER stress occurs when misfolded proteins accumulate in the lumen of the ER (Lin et al. 2008). The cell responds to these misfolded proteins with the conserved unfolded protein response (UPR), which reduces the protein load in the ER and increases the capacity of the ER to handle unfolded proteins (Ron and Walter 2007). The UPR involves a significant transcriptional response that activates a large number of UPR target genes. There are 3 different branches of the UPR: ATF6, IRE1, and PERK, each with their own unique target genes and roles (Walter and Ron 2011).

Despite being a well-conserved process vital to basic cellular function, the ER stress response displays transcriptional variability, measured through microarray and RNA-seq studies, dependent on genetic background in *Drosophila*, mouse, and humans (Dombroski et al. 2010; Chow et al. 2013, 2015; Russell and Chow 2022). In the case of the *Drosophila* study, the transcriptional variability also led to a variable physiological outcome which was measured through survival time (Chow et al. 2013). The ER stress response is a critical component of various diseases, including neurodegeneration, diabetes, and cancer (Katayama et al. 1999; Imai et al. 2000; Özcan et al. 2004; Carrasco et al. 2007; Laybutt et al. 2007; Saxena et al. 2009; So et al. 2009). The transcriptional variability observed in the UPR can significantly influence physiological and disease outcomes in conditions with a prominent ER stress component. A better understanding of the factors contributing to the transcriptional variability of the UPR provides valuable insights into its role in disease development and the modification of disease severity.

One of the potential factors underlying the transcriptional variability that leads to altered transcript levels is genetic variants that affect regulatory mechanisms. This could have a significant influence on the cellular responses to ER stress. Genetic variants can affect transcriptional levels through regulatory mechanisms in either a *cis*- or *trans*- manner. Previous work has identified hundreds of genes that were impacted by *cis*- and *trans*-regulatory mechanisms which influence transcript levels under ER stress (Chow et al. 2015; Russell and Chow 2022). There are many types of *cis*-regulatory mechanisms that can impact gene expression, such as promoters, enhancers, silencers, and insulators, many containing binding sites for transcription factors (TFs).

TFs bind to a preferred DNA sequence, known as a “binding motif” (Boeva 2016). A genetic variant within the specific DNA sequence that the TF recognizes could impact binding specificity, binding dynamics, and even DNA methylation profiles (Johnston et al. 2019; Martin-Trujillo et al. 2020). Natural genetic variation in multiple TF binding sites (TFBSs), and subsequent altered TF binding, can lead to widely different expression profiles among individuals (Johnston et al. 2019; Tseng et al. 2021). Common *cis*- variants affect approximately 30% of expressed genes, and it is thought that polymorphisms within TFBSs that change protein binding dynamics are a major contributing factor to this variation (Ge et al. 2009; Wong et al. 2011).

Differences in TF binding activity can alter expression levels and impact human phenotypic variability and ultimately lead to disease. This has been demonstrated for processes such as immunity and hematopoiesis. Variants within the binding sites of the NF-κB family of TFs can lead to variable binding of TFs, which results in variable expression patterns. This can partly explain the phenotypic diversity in human immune responses (Kasowski et al. 2010; Wong et al. 2011). Mutations involved in human erythroid disorders disrupt GATA1 binding, a known hematopoietic TF (Wakabayashi et al. 2016). These are just a few examples of how variation in TFBSs can lead to a variable transcriptional response which can underlie disease. This potential motivates our study of variation in the ER stress response.

The 3 branches of the UPR are part of a signaling cascade that culminates in 3 main TFs, ATF4, XBP1, and ATF6, eliciting a large transcriptional response to misfolded proteins (Ron and Walter 2007). ATF6 binds in conjunction with another TF, NFY, to 2 different binding motifs that differ in spacing and orientation. These 2 sites are known as ER stress response element I (ERSEI) and ERSEII (Yoshida et al. 1998; Kokame et al. 2001). The binding motif for XBP1 is known as the UPR element (UPRE) (Mori et al. 1992, 1996). The binding motif for ATF4 in this study will simply be referred to as “ATF4.” Natural genetic variation present within the binding sites of these 3 TFs could lead to variable expression patterns and could partially underlie the variable response to ER stress that has been seen in multiple organisms (Dombroski et al. 2010; Chow et al. 2013, 2015; Russell and Chow 2022).

To study how genetic variation might impact ER stress–related TFBSs, we addressed 4 questions. First, we sought to determine the locations of the binding sites for the 3 main ER stress TFs, XBP1, ATF6, and ATF4. To address this, we created a pipeline to predict all possible genome-wide binding sites for these factors. Second, we aimed to identify which of these predicted binding sites are more likely to be true binding sites. To achieve this, we implemented multiple filtering steps based on previous experimental and computational data to identify binding sites that are more likely to be genuine. Third, we identified the positions within these binding sites that exhibit the most variability. For this inquiry, we relied on human genetic, human disease, and evolutionary conservation data to identify the positions that display the highest variability in these putative binding sites. And fourth, we sought to understand how variation in these binding sites affects expression levels. To answer this question, we performed *cis*-expression quantitative trait locus (eQTL) analyses on expression data from a set of unrelated human cell lines exposed to both control and ER stress conditions. Notably, to the best of our knowledge, this is the first *cis*-eQTL analysis performed on human cell lines after the induction of ER stress. Our findings provide compelling evidence for how natural genetic variation within the human population can lead to a diverse ER stress response. Understanding the genomic locations of these binding sites, the prevalence of genetic variation within them, and how this variation can alter gene expression is vital in determining how TF binding activity can underlie variable susceptibility in human diseases with an ER stress component.

Additionally, while this work is focused on the 3 main TFs of the ER stress response, it can be applied to other TFs and other processes. As stated previously, TF binding variability can be linked to many different diseases, indicating the value of investigating this further. The responses to the first 3 main questions of this study can be adapted and applied to other TFs and data sets. This study can provide a resource in 3 different ways: (1) description of a pipeline for identifying potential TFBSs, (2) a list of potential binding sites of the 3 main TFs of the ER stress response which includes putative novel ER stress genes, and (3) a list of variants within TFBSs that we show, through *cis*-eQTL analysis, to have an effect on gene expression after the induction of ER stress.

## Materials and methods

### Motif scan across human genome

We used PWMScan (https://epd.expasy.org/pwmtools/pwmtools/pwmscan.php) to identify positions of the 4 predicted binding sites across the human genome (GRCh37/hg19) (Ambrosini et al. 2018). Overlapping matches were included. The *P*-value cutoffs used for each motif were as follows: ERSEI: *P* ≥ 1.6 × 10^−05^; ERSEII: *P* ≥ 1.6 × 10^−05^; UPRE: *P* ≥ 1.2 × 10^−04^; and ATF4: *P* ≥ 1.9 × 10^−05^. These *P*-values allow one “wobble” base for the next most likely base at that position. The input for PWMScan for each motif was a letter probability matrix (LPM) generated with data from JASPAR (http://jaspar.genereg.net/). In an LPM, each column represents a nucleotide in a motif sequence (A, C, G, and T, respectively), and each row represents a nucleotide's position in the motif. The LPM is constructed by using experimental data, and each number represents the probability of a specific nucleotide occupying that position at a known binding location of the TF of interest. For example, a value of 1 in the first column indicates that an A nucleotide is found at that specific position 100% of the time the TF is bound to DNA. A row of 4 entries of 0.25 indicates that each nucleotide has an equal probability of being found at that position when a TF is bound. LPMs for our TFs of interest are detailed in Supplementary Table 1. The results of this search resulted in 224,244 motifs (ERSEI: 20,565; ERSEII: 18,410; UPRE: 60,202; and ATF4: 125,067) which are listed in Supplementary Table 1.

### Gene proximity filtering

Lists of protein-coding genes were obtained from Ensembl (GRCh37) (https://grch37.ensembl.org/index.html). Filtering of motifs based on proximity to a gene was performed using the window tool of bedtools v2.28.0 (Quinlan and Hall 2010), which searches for a predicted binding motif within a specified window of a protein-coding gene. For example, when a 2 kb window was set, there was a hit if there was a predicted binding motif 2 kb upstream of the gene, within a gene, or 2 kb downstream of the gene. The cutoff value of 2 kb is based on the accepted average distance between *cis*-regulatory elements, such as promoters and enhancers, from the transcriptions start site (TSS) (Bulyk 2004; Blanchette et al. 2006; Maderazo et al. 2022). While we used a value of 2 kb, we recognize the actual distance can vary greatly.

### Human conservation scores

To determine the conservation of a region of DNA, namely the predicted binding site, we utilized PhastCons scores (Felsenstein and Churchill 1996; Siepel et al. 2005). To calculate this, we used the bigWigAverageOverBed tool from Kent utilities (Kent et al. 2010). We only included motifs in the conservation filtering step that had a PhastCons score provided for each base of the given motif. As in previous publications (Sun and Yu 2019), we used a cutoff of 0.5 to define a conserved region. This value is somewhat arbitrary, and the use of other values would lead to slightly different results. To determine the average conservation of each base of each motif, we utilized PhyloP scores (Pollard et al. 2010). To calculate the PhyloP score of each position, we similarly used the bigWigAverageOverBed tool from Kent utilities (Kent et al. 2010).

To determine whether any positions in the TF binding motif were significantly more or less conserved than the other positions, we calculated the average conservation score at each position using PhyloP Scores. We then performed an analysis of variance (ANOVA) test to determine whether there were significant differences in conservation scores between positions. If the ANOVA showed a significant difference, we performed a post hoc Tukey’s honest significant difference (HSD) test to identify which positions differed significantly from each other.

In addition, we performed a separate post hoc Tukey’s HSD test to compare the conservation score at positions that had higher Genotype-Tissue Expression (GTEx) *cis*-eQTLs to the conservation scores at all other positions combined. We used a significance level of 0.05 for all tests and adjusted the *P*-values for multiple comparisons as necessary. We did not directly compare the 4 different motifs to each other, and each ANOVA and subsequent *P*-value corrections were treated as separate entities, making another correction unnecessary. As a result, the interpretations and conclusions for each motif analysis remain confined within each respective independent analysis. All statistical analyses were performed using R version 4.1.2.

### GTEx data set

We used data provided by GTEx (https://gtexportal.org/home/) to investigate tissue-specific genetic variants that impact expression levels. We used GTEx v7 data that were aligned to build GRCh37 of the human genome. Within this data set, there were *cis*-eQTL data for 48 tissues. For each tissue, we searched for overlap of significant *cis*-eQTLs with our putative TFs across the human genome using bedtools intersect v2.28.0 (Quinlan and Hall 2010).

To determine whether the observed counts of *cis*-eQTLs at a specific position differed significantly from the counts for all the other positions combined, assuming equal counts across all positions, a chi-squared test of goodness-of-fit was performed. Specifically, the observed counts for the target position were compared to the expected counts based on the total number of variants across all positions, and a chi-squared test statistic was calculated to assess the level of difference between the observed and expected counts. The significance level was set at 0.05, and a Bonferroni correction was applied to adjust for multiple hypothesis testing. All statistical analyses were performed using R version 4.1.2, and the chi-squared test was conducted using the “chisq.test” function in R.

### Random motif data set generation

To make a comparison for overlap between human data sets and predicted motifs, we created a data set representing randomly generated regions of the genome, which represent the same number of predicted motifs (224,244) across the genome. These regions are also the same length and number as the respective motif they are simulating (ERSEI: length 10, count 20,565; ERSEII: length 10, count 18,410; UPRE: length 10, count 60,202; and ATF4: length 8, count 125,067). ERSEI and ERSEII are length 10 given that the middle nucleotides (9 for ERSEI; 1 for ERSEII) have no impact on TF binding and were not included in the overlap comparison of predicted motifs. Only the first 5 and last 5 nucleotides were included in the overlap comparison; thus, a length of 10 was used. To generate these random regions across the human genome (GRCh37/hg19), we used the random command of bedtools v2.28.0 (Quinlan and Hall 2010). We then used the intersect command of bedtools to assess overlap between the randomly generated regions and the data set of interest. To assess statistical significance, random generation of genomic regions was repeated 9,999 times. To calculate an unbiased estimate of the *P*-value for the comparison of simulated overlap between the real overlap of our putative motifs and the data set of interest, we used this equation: (#{n* >= *n*[real]} + 1)/(*N* + 1). N* represents the number of overlaps between random motifs and the data set of interest, *n*[real] represents number of real overlaps between putative motifs and the data set of interest, and *N* represents the number of permutations. A Bonferroni correction was applied to adjust for multiple hypothesis testing.

### Overrepresentation of benign ClinVar variants at positions within a binding motif

Variant enrichment analysis was performed to assess whether there was a significant overrepresentation of benign variants at specific positions within the 4 binding motifs. For each binding motif, the total number of benign and pathogenic variants across the motif was obtained. The probability of a variant being benign was calculated as the ratio of the total number of benign variants to the total number of variants (benign and pathogenic) across the motif.

For each position within the motif, the number of benign and pathogenic variants was counted. A one-sided binomial test was then used to calculate the probability of observing at least as many benign variants as observed, assuming a null hypothesis of random distribution of benign and pathogenic variants. The significance level was set at 0.05, and the Bonferroni correction was applied to adjust for multiple hypothesis testing.

The results were reported as the *P*-value for each position within the binding motif. A low *P*-value (<0.05 after Bonferroni correction) indicated a significant overrepresentation of benign variants at that position, suggesting a potential functional importance of that position within the binding motif.

### *Cis*-eQTL analysis on the Centre d'Etude du Polymorphisme Humain individuals

Expression data for cell lines were derived from individuals included in the Centre d'Etude du Polymorphisme Humain (CEPH; Human Polymorphism Study Center) and were obtained from GEO (https://www.ncbi.nlm.nih.gov/geo/) (accessions GSE36911 and GSE36910) (Dausset et al. 1990). These data sets were generated from 2 previous studies that evaluated gene expression of these cell lines after the induction of ER stress using tunicamycin (TM) (Dombroski et al. 2010; Nayak et al. 2014). TM is a well-established drug that induces ER stress by inhibiting *N*-linked glycosylation (Tkacz and Lampen 1975). CEPH DNA samples were whole-genome sequenced at an average depth of 30× using Illumina paired-end technology (Sasani et al. 2019). Variant call information (VCFs) is available at dbGaP under controlled access, with accession phs001872.v1.p1. For every CEPH individual for whom we had both expression and sequencing data (110 individuals), we ran a *cis*-eQTL analysis using FastQTL (Ongen et al. 2016). FastQTL performs linear regressions between genotypes and molecular phenotypes with or without covariates to find the best nominal association for each phenotype. No covariates were defined in this analysis. We defined the mapping window as 1 Mb up- and downstream of the TSS of a gene. As suggested by FastQTL documentation, we used false discovery rate (FDR) correction to control for the number of false positives, with an FDR-adjusted *P*-value cutoff of *P*-adj < 0.001.

To compare the effect sizes of significant *cis*-eQTLs found in TM conditions with the respective *cis*-eQTLs in control conditions, whether found to be significant or not, we followed a previously described method for obtaining effect sizes and standard error from FastQTL output (Urbut et al. 2018). To compare the effect sizes, we performed a Welch modified 2-sample *t* test and considered the difference between effect sizes of the TM and control to be significant if the 90% confidence interval of the mean difference did not cross 0. We performed this same analysis for significant *cis*-eQTLs found in control conditions and compared to the respective *cis*-eQTLs in TM conditions. We then identified which significant *cis*-eQTLs overlapped putative binding sites by using bedtools intersect as described previously.

### Human data sets

To study clinically relevant variants in the human population, we used data provided by ClinVar (https://www.ncbi.nlm.nih.gov/clinvar/) (Landrum et al. 2018). To best represent clinically relevant variants, we only investigated 143,687 ClinVar entries categorized as “pathogenic” or “likely pathogenic.” We then compared them to “benign” and “likely benign” variants.

The middle nucleotides of ERSEI and ERSEII are not necessary for TF binding (Yoshida et al. 2000). Therefore, we only counted SNPs that were within the first 5 and last 5 nucleotides of ERSEI and ERSEII. While the nucleotides themselves can be variable, the number of them is important in order to maintain correct spacing between the first and last 5 nucleotides (Yoshida et al. 2000). Thus, we included any deletions or insertions that were at any position within an ERSEI and ERSEII motif as these would alter the spacing of the binding sites of ATF6 and NFY, ultimately impacting functionality of ERSEI and ERSEII.

### Enrichment analysis

All gene ontology analyses and pathway enrichment analyses were performed with DAVID 2021 (Huang et al. 2009; Sherman et al. 2022). We used the Benjamini–Hochberg method for adjusting for multiple testing, with a *P*-adj cutoff of 0.05. The Benjamini correction, which is widely used in enrichment analysis (Benjamini and Hochberg 1995; Lewin and Grieve 2006), strikes a balance between statistical power and control of false positives.

## Results and discussion

### Genome-wide identification of binding motifs

There is substantial functional characterization of the TFs involved in the ER stress response. Using these data and the subsequently validated binding site sequences of these TFs, we created a computational pipeline to identify all possible binding sites. ChIP-seq experiments are the most direct way in which TFBSs are identified (Farnham 2009; Park 2009; Furey 2012). While this is still an effective method to identify TFBSs, one of the major downfalls is the introduction of experimental bias. The binding of a TF to DNA is subject to many different factors, such as cell type, developmental stage, chromatin accessibility, and stress state (Slattery et al. 2014). To get a complete picture of every possible UPR-related TFBS in the human genome, one would have to perform ChIP-seq on an astronomical number of samples from many different cell types at different developmental stages or cellular stressors; however, one constant within every cell type and every condition is genome sequence. We took advantage of this by identifying experimentally validated binding sequences throughout the human genome to create an experimentally unbiased list of potential TFBSs.

**Fig. 1.**
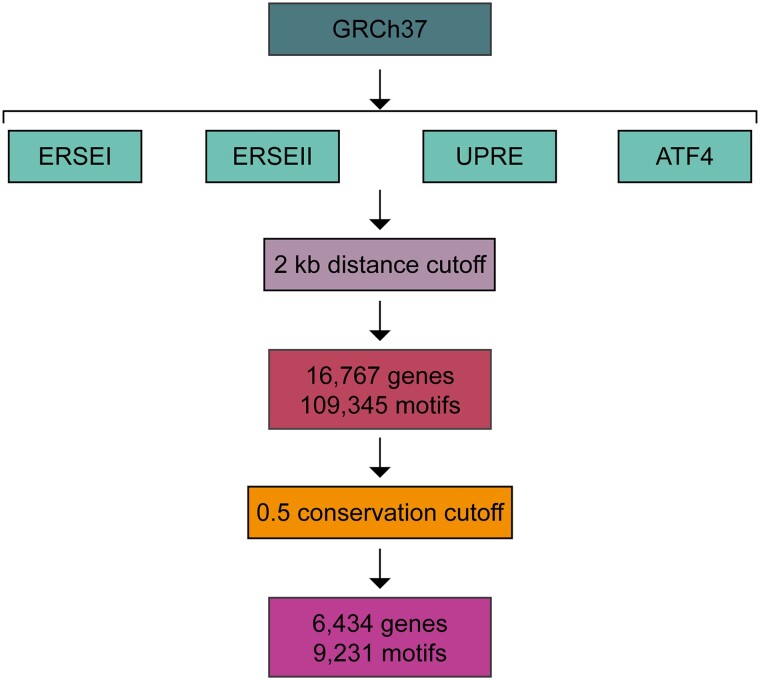
Pipeline for identifying TFBSs. Pipeline describing the search for ERSEI, ERSEII, UPRE, and ATF4 binding motifs across the human genome (GRCh37). Binding sites were first filtered on proximity to a protein-coding gene. Total number of motifs and total number of genes with a binding site motif within a 2 kb window are reported. The resulting sites were then filtered based on PhastCons conservation score. Total number of motifs with a PhastCons score > 0.5 and corresponding total number of genes are reported.

To identify predicted binding sites of UPR TFs, we searched genome-wide (GRCh37) for 4 literature-supported motifs: ERSEI (CCAAT-N9-CCACG), ERSEII (ATTGG-N-CCACG), UPRE ((C/A)CACGTCA), and ATF4 ((A/C)TGA(T/C)GCAA(T/C)) (Fig. 1). To accommodate for base-pair variability within a binding motif, we utilized a LPM search instead of a consensus sequence–based search (Supplementary Fig. 1 and Tables 1 and 2). The genome-wide search for predicted binding sites produced 224,244 candidate motifs.

ERSEI, ERSEII, UPRE, and ATF4 binding sites are found in *cis*- near known ER stress genes (Mori et al. 1992, 1996; Yoshida et al. 1998; Kokame et al. 2001; Siu et al. 2002). Because this pattern likely holds true for other target genes, we filtered on distance to nearest protein-coding gene (2 kb) and only included TFBSs that met these criteria (Fig. 1; Supplementary Table 3). Applying this distance criterion narrowed down results from 224,244 motifs to 109,345 motifs, corresponding to 16,767 unique protein-coding genes (Supplementary Table 2). As a proof of principle, genes with known roles in the ER stress response, *HERP*, *BIP*, *ERO1L*, and *CHOP* (Kokame et al. 2001; Ma et al. 2002; Baumeister et al. 2005; Wang and Ouyang 2011), were identified in this set of genes.

Cross-species conservation can also help to identify potential TFBSs. The use of conservation scores as a filter is particularly advantageous, as it remains consistent regardless of cell type, tissue type, developmental stage, or environmental stressors. Conservation is a useful filter because a region of DNA that is conserved is more likely to serve a vital function (Kimura and Ota 1974; Pennacchio et al. 2006; Nitta et al. 2015). We used PhastCons as our measure of conservation as it measures the probability that each nucleotide belongs to a conserved element based on multiple alignments (0–1 score) (Felsenstein and Churchill 1996; Siepel et al. 2005). We used a cutoff of 0.5 (50% chance that a nucleotide belongs to a conserved element) and only included motifs that were completely covered by PhastCons scores. This further narrowed down our list of motifs from 109,345 to 9,231 (Fig. 1; Supplementary Tables 4 and 2), corresponding to 6,434 protein-coding genes.

Gene ontology (GO) enrichment of these 6,434 genes revealed the most significant enrichment was for positive regulation of transcription from RNA polymerase II promoter (GO:0045944, *q* = 1.3 × 10^−21^) (Supplementary Table 5). This enrichment for genes that increase transcription may indicate a signaling cascade that starts with the 3 main UPR TFs targeting other transcriptional activators, leading eventually to the large transcriptional response of the UPR. To determine if the number of known ER stress genes in this list exceeded the number expected by chance, we next focused on pathway enrichment. We found that protein processing in ER is significantly enriched (KEGG pathway entry: hsa04141, *q* = 2.9 × 10^−5^) (Supplementary Table 5). This pathway contains the genes responsible for proper protein glycosylation and transport in the ER, as well as proper degradation of misfolded proteins in the ER. Our list of 6,434 genes contains many genes within this pathway, such as the 4 mentioned previously (*HERP*, *BIP*, *ERO1L*, and *CHOP*), as well as other canonical ER stress genes such as *PERK*, *IRE1*, *HSP40*, *HRD*1, and *GRP94*. In fact, there is higher enrichment for this pathway in the more filtered list (6,434 genes, *q* = 2.9 × 10^−5^) compared to the less filtered list (16, 767 genes, *q* = 0.023).

These 6,434 genes are the product of a computational pipeline to identify potential binding sites of the 3 main TFs of the UPR, and each is a potential UPR target gene. While this method removes the experimental biases mentioned earlier, potential computational biases are introduced, such as initial motif sequence selection and subsequent filtering steps. However, while both experimental methods and our computational method have their respective biases, they complement each other.

### Tissue-specific regulatory variation within binding motifs

Given that these regions represent potential binding sites for TFs, we hypothesized they would have an impact on gene expression levels. To test this, we utilized the online GTEx data set (GTEx Consortium 2017). The GTEx data set contains gene expression, genotype, and eQTL data for 54 tissues from nearly 1,000 nondiseased individuals. An eQTL is a region of DNA that affects the expression of a gene. GTEx contains many eQTL for different genes across all the tissues. We hypothesized that some of these eQTLs overlap UPR TF motifs and could impact gene expression if there is differential binding or activity.

To investigate this, we first determined how many significant GTEx *cis*-eQTLs localized to the predicted TFBSs. We imposed no distance thresholds (minimum distance to a gene), as the GTEx data set already uses one for their eQTL analysis (mapping window defined as ±1 Mb of the TSS). We found that the number *cis*-eQTLs localized to the predicted TFBSs across the 48 tissues tested is strongly dependent on tissue type (Supplementary Tables 6 and 7). Next, we compared the overlap between *cis*-eQTLs and predicted motifs to the overlap of *cis*-eQTLs and randomly generated regions across the human genome to determine if *cis*-eQTLs are enriched within predicted motifs. We created a data set of randomly selected positions across the human genome that corresponded to the same length and number of the predicted motifs. As before, we intersected the randomly generated regions with *cis*-eQTLs across the 48 different tissues and quantified the overlaps (Supplementary Table 7). For all 4 binding motifs, across all tissues, there is a significantly higher overlap for GTEx *cis*-eQTLs with predicted motifs than would be expected by random chance (Supplementary Table 7). For example, 938 overlaps between lung *cis*-eQTLs and randomly generated regions are expected by chance. However, we observed 2,642 overlaps between lung *cis*-eQTLs and predicted TFBS (*P*-adj = 0.0048).

**Fig. 2.**
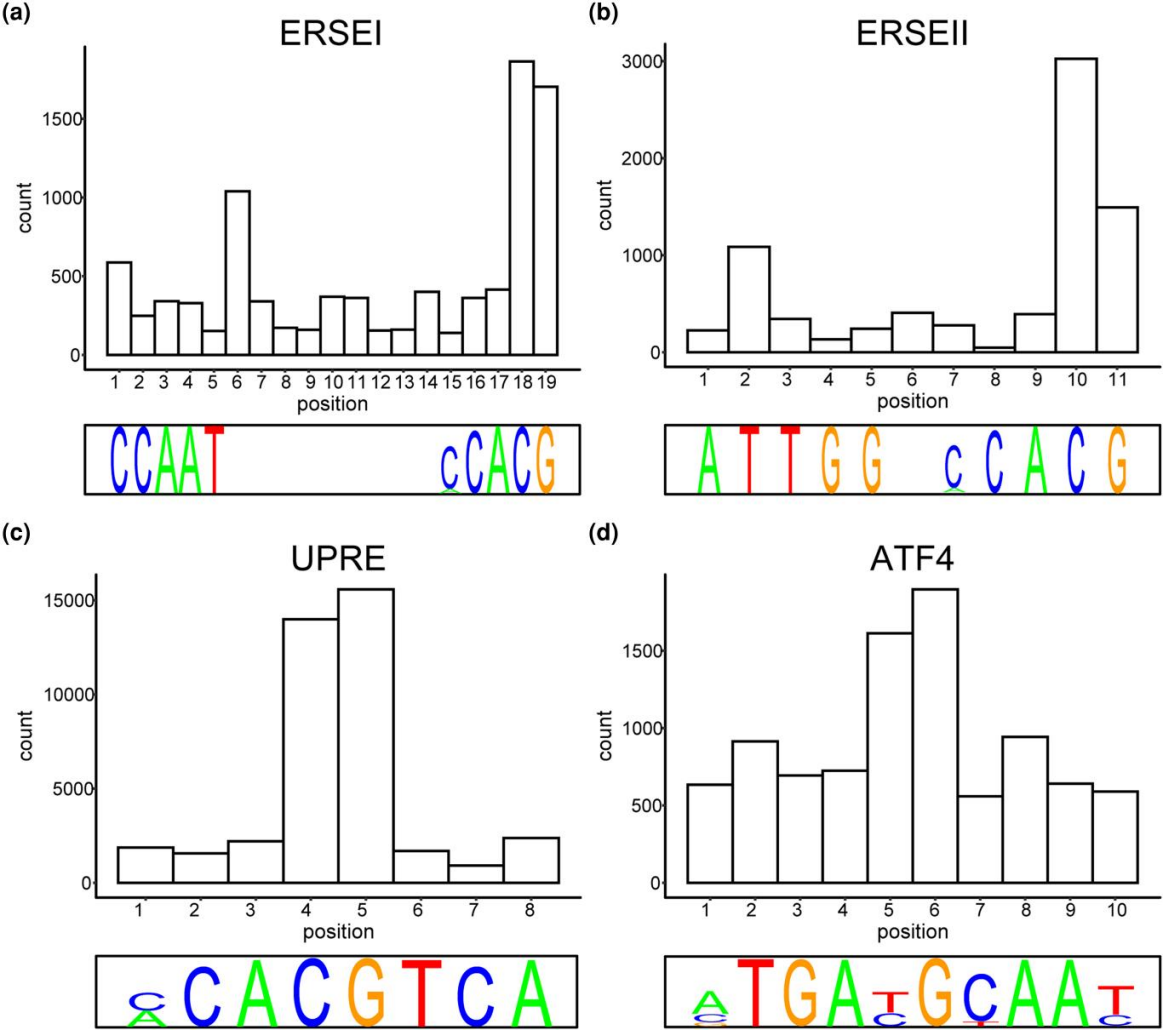
Certain positions within a binding motif harbor more GTEx *cis*-eQTLs than others. Histograms plotting number of significant GTEx *cis*-eQTLs that fall within each position of the 4 binding motifs assayed, ERSEI a), ERSEII b), UPRE c), and ATF4 d). Representative LPM for each of the binding motifs is displayed on the bottom of the histogram.

**Fig. 3.**
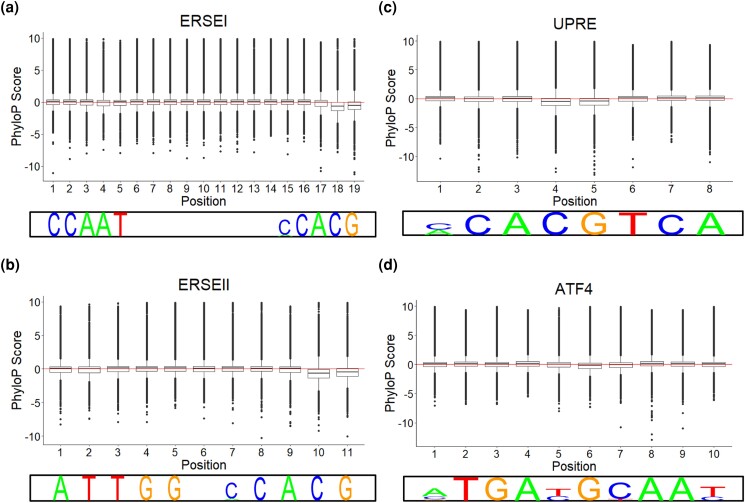
Average PhyloP scores of each motif position. The average PhyloP score for each position of each binding motif assay is plotted, for ERSEI a), ERSEII b), UPRE c), and ATF4 d). The line plotted at 0 is to denote neutral evolution.

After combining all tissue data, we identified where each *cis*-eQTL is localized in in each respective binding motif. For each binding motif, we found that certain positions harbor more *cis*-eQTLs than expected by chance (ERSEI: positions 18 and 19; ERSEII: positions 10 and 11; UPRE: positions 4 and 5; ATF4: positions 5 and 6) (Fig. 2) (*χ*^2^*P*-adj: ERSEI: 2.14 × 10^−200^, 4.01 × 10^−166^; ERSEII: 0, 5.07 × 10^−74^; UPRE: 0, 0; ATF4: 1.93 × 10^−48^, 1.31 × 10^−87^). To further investigate these positional *cis*-eQTL hotspots, we determined the conservation of each position and found that most of the hotspots for *cis*-eQTLs were far less conserved than the other positions (Fig. 3) (Tukey HSD *P*-adj: ERSEI positions 18 and 19: <2.2 × 10^−16^; ERSEII positions 10 and 11: <2.2 × 10^−16^; UPRE positions 4 and 5: <2.2 × 10^−16^; ATF4 positions 5 and 6: <2.2 × 10^−16^).

In each of the motifs, the GTEx *cis*-eQTL hotspots are CpG dinucleotides and most of the variation is caused by C→T transitions (Supplementary Fig. 2). CpG dinucleotides are rare throughout the genome, occurring at only one-fifth of the ∼4% expected frequency (International Human Genome Sequencing Consortium 2001; Hodgkinson and Eyre-Walker 2011). The rate of mutation of CpGs is elevated by ∼30-fold relative to the average rate of mutation in great apes (Hwang and Green 2004). Despite the underrepresentation of CpG dinucleotides genome wide, they comprise ∼25% of all mutations in humans (Fryxell and Moon 2005). This high mutation rate is due to deamination of methylated cytosines, resulting in a C→T transition (Coulondre et al. 1978), which is exactly what we observed at the *cis*-eQTL hotspots.

### Clinically relevant variants present within TF binding motifs

We next investigated the relevance of variants in these binding motifs to human disease. Many clinically pathogenic variants are recorded in ClinVar (Landrum et al. 2018), which includes variants ranging from known benign to known pathogenic. To best represent clinically relevant variants, we only investigated those categorized as “pathogenic,” “likely pathogenic,” “benign,” or “likely benign.” We quantified the overlap between these variants and our predicted binding motifs (Supplementary Table 8). Pathogenic and likely pathogenic variants have data supporting the impacted gene; therefore, we did not filter our potential binding motifs by gene proximity. There were 213 (of 143,687; 0.14%) pathogenic or likely pathogenic ClinVar variants that fell within a predicted binding motif (Supplementary Table 8). We included an additional 7 overlaps not within a binding site, because the variants were indels located between the 2 binding sites of ERSEI and ERSEII which would alter spacing and TF binding activity. The positions of the ClinVar variants within each respective motif are shown in Supplementary Fig. 3.

We then compared the overlap of predicted motifs and ClinVar variants to the overlap with randomly generated regions to obtain the number of overlaps expected by chance (Supplementary Table 9). ERSEI, ERSEII, and UPRE have a greater overlap with pathogenic variants than would be expected by chance (ERSEI: *P*-adj = 0.0048; ERSEII: *P*-adj = 0.0052; UPRE: *P*-adj = 0.0021). ATF4 does not have a greater overlap than expected by chance (*P*-adj = 1), suggesting that ATF4 variants are either less likely to be pathogenic or so damaging that they are seldom found in a population.

**Fig. 4.**
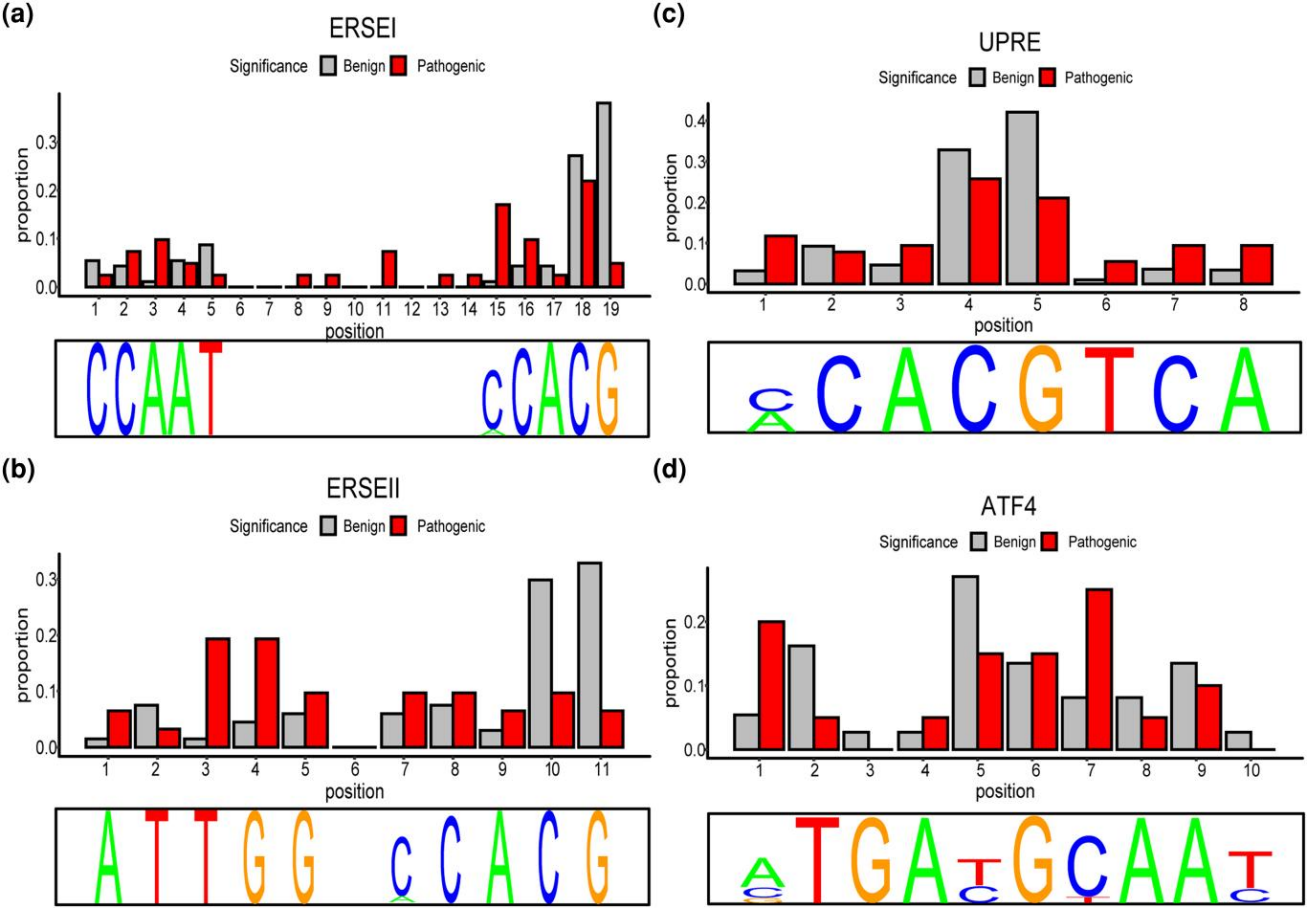
Comparison of pathogenic and benign ClinVar variants at each position. The proportion of pathogenic (pathogenic variants at a given position/all pathogenic variants) vs benign (benign variants at a given position/all benign variants) ClinVar variants at each position for ERSEI a), ERSEII b), UPRE c), and ATF4 d).

We next performed the same analysis on benign variants found in ClinVar, and we found 695 (of 308,497; 0.22%) benign ClinVar variants that overlapped with predicted motifs (Supplementary Table 8). The position of a benign ClinVar variant within a respective motif is summarized in Supplementary Fig. 4. By comparing benign variants to pathogenic variants and their positions within the binding motif, we can determine which base positions are more likely to cause disease when mutated. We performed this comparison for each base position for each motif (Fig. 4; Supplementary Figs. 5 and 6).

While we expected to observe an equal distribution of benign and pathogenic variants, we found that certain positions within the sequence had a higher frequency of pathogenic variants than benign variants. For instance, the ClinVar database revealed that position 15 of ERSEI contained 7 pathogenic variants and only one benign variant (Supplementary Fig. 5 and 6). To account for the difference in the total number of benign and pathogenic variants, we analyzed the proportion of each type of variant. Our results revealed that some positions had a higher proportion of pathogenic variants, such as position 3 in both ERSEI and ERSEII, suggesting that mutations at these positions may be more likely to result in pathogenic outcomes (Fig. 4). Interestingly, nearly every CpG position noted previously has proportionally more benign variants than pathogenic, with ATF4 as the exception. In fact, for the CpG sites in the other 3 motifs, there is a significantly higher representation of benign variants than expected at the given position (ERSEI position 18: *P*-adj = 3.2 × 10^−10^, position 19: *P*-adj = 3.7 × 10^−19^; ERSEII position 10: *P*-adj = 2.5 × 10^−05^, position 11: *P*-adj = 1.4 × 10^−06^; UPRE position 4: *P*-adj = 4.6 × 10^−30^, position 5: *P*-adj = 5.1 × 10^−57^). This may suggest that these CpG sites, while highly mutable, are less likely to cause disease.

### *Cis*-eQTL analysis on a large human population data set experiencing ER stress

Previous studies have shown that the majority of regulatory mechanisms and eQTLs act in a context-dependent manner (Chow et al. 2015; Russell and Chow 2022). Based on this, the GTEx data, in healthy nondisease tissues, would partially differ from eQTLs that are found under ER-stressed conditions. To identify ER stress–relevant eQTLs, we performed a *cis*-eQTL analysis on human cells exposed to ER stress.

We performed a *cis*-eQTL analyses using previously published gene expression data, under control and ER stress conditions, in human cell lines from 131 unrelated individuals (Dombroski et al. 2010; Nayak et al. 2014). ER stress was induced by exposing the cells to a well-established ER stress–inducing drug, TM (Tkacz and Lampen 1975). The cell lines were derived from individuals in the CEPH (Human Polymorphism Study Center) (Dausset et al. 1990). While this cohort is composed of large families, the samples in the ER stress study were unrelated individuals sampled from different families. Because whole-genome sequence data are available for these individuals (Sasani et al. 2019), we paired expression data under control and ER stress conditions with sequencing data to identify ER stress–specific *cis*-eQTLs (Supplementary Table 10).

Under control conditions, we found 836,115 *cis*-eQTLs with a cutoff of *P*-adj < 0.001 (FDR correction). Under TM conditions, we found 823,507 *cis*-eQTLs with a cutoff of *P*-adj < 0.001. The majority of these *cis*-eQTLs are unique to a given condition (370,895 shared; 465,220 unique to control; 452,612 unique to TM) (Supplementary Fig. 7 and Table 10). Additionally, we compared the effect sizes of the significant *cis*-eQTLs in either condition (ER stress vs control) to the respective *cis*-eQTL in the other condition to get another representation of condition-specific *cis*-eQTLs. We found that of the 823,507 significant *cis*-eQTLs, 293,065 (35.58%) had a different effect size under control conditions. Alternatively, of the 836,115 significant control *cis*-eQTLs, 301,587 (36.07%) had a different effect size under ER stress conditions. The large number of *cis*-eQTLs that are unique to TM-induced ER stress conditions highlights just how large and complex the ER stress response is. This list of significant ER stress–specific *cis*-eQTLs represents a valuable resource for the exploration of the genetic components underlying interindividual variation in the ER stress response. These *cis*-eQTLs correspond to 14,973 genes unique to TM, 4,686 genes unique to control, and only 142 genes seen in both conditions (Supplementary Table 11). Only genes with *cis*-eQTLs unique to TM conditions showed any functional enrichment. The most enriched category was regulation of transcription, DNA templated (Supplementary Table 11).

**Fig. 5.**
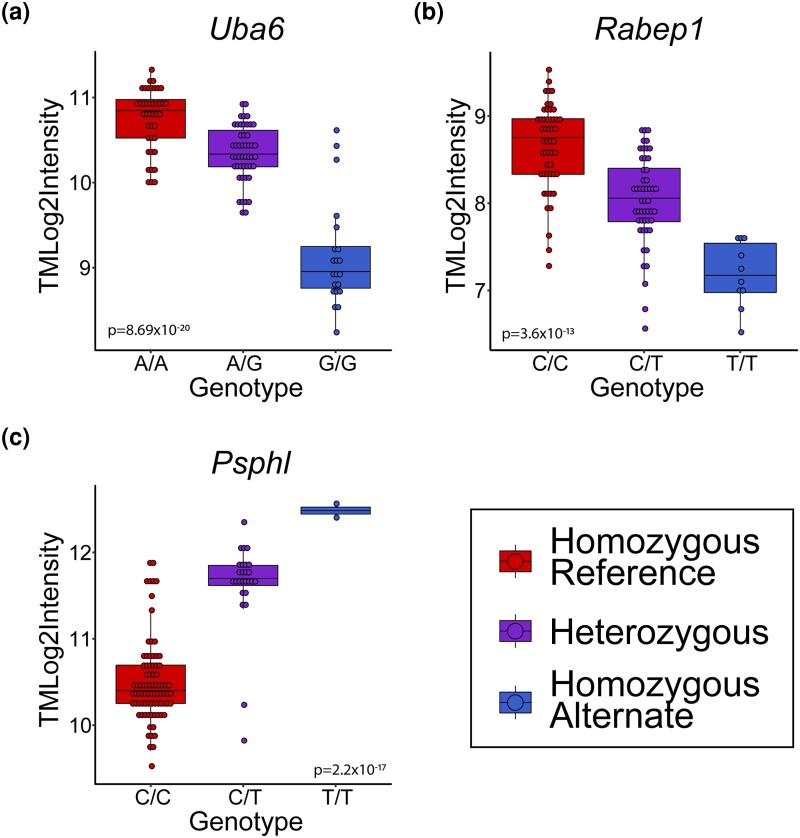
Significant *cis*-eQTLs that fall within predicted TFBSs. Log2 intensity under TM conditions is plotted for each genotype for the genes *Uba6* a), *Rabep1* b), and *Psphp1* c). *P*-adj value is the FDR-corrected *P*-value generated by FastQTL.

We next determined if ER stress–specific *cis*-eQTLs are localized to UPR TFBSs. We intersected the 823,507 significant *cis*-eQTLs under TM conditions with our putative TFBSs and investigated the overlaps (Supplementary Table 12). These *cis*-eQTLs represent binding sites with variants that affect gene expression only under ER stress conditions and not control conditions, highlighting how these potential ER stress genes would have been missed if only studying control conditions. The most significant ER stress–specific *cis*-eQTL was an ATF4 binding motif approximately 900 kb upstream of the gene *Ubiquitin-like Modifier Activating Enzyme 6* (*UBA6*) (*P*-adj = 8.69 × 10^−20^). Individuals who are homozygous for the alternate allele have, on average, lower levels of *UBA6* expression when exposed to TM (Fig. 5a) (average TMLog2Intensity: homozygous reference (A/A): 10.75; heterozygous (A/G): 10.34; homozygous alternate (G/G): 9.13). The decreased expression could be due to decreased affinity of ATF4 to this variable binding site and subsequent decreased expression levels under ER stress conditions. This is supported by the fact that individuals who are G/G have the lowest fold change of *UBA6*, indicating that even when taking control levels into account, the G/G genotype is less responsive to ER stress. The alternate allele (G) (allele frequency = 0.37) is also the ancestral allele. *UBA6* is an ubiquitin-activating enzyme that initiates ubiquitination by ATP-dependent activation of ubiquitin. The human genome encodes 2 ubiquitin-activating enzymes, the canonical E1 enzyme *UBA1* and the noncanonical E1 enzyme *UBA6* (Schulman and Harper 2009; Liu et al. 2017). Ubiquitination plays a critical role in the regulation of the cell's proteome (Swatek and Komander 2016). Therefore, there is potential for *UBA6* to be a UPR target gene, given its role in protein regulation.

The most significant *cis*-eQTL in an ERSEII motif is for *RABEP1* (*Rabaptin*, *RAB GTPase Binding Effector Protein 1*) (*P*-adj = 3.6 × 10^−13^) (Fig. 5b). *RABEP1* expression is associated with a SNP located within an ATF6-binding motif approximately 40 kb downstream of RABEP1. *RABEP1* acts as a linker protein that is involved in endocytic membrane fusion and membrane trafficking of recycling endosomes (Stenmark et al. 1995). Crosstalk between the ER and endosomes impacts protein trafficking (Rowland et al. 2014). Individuals homozygous for the alternate allele have significantly lower expression levels of *RABEP1* under ER stress conditions than those who are homozygous for the reference allele (Fig. 5b) (average TMLog2Intensity: homozygous reference (C/C): 8.95; heterozygous (C/T): 8.21; homozygous alternate (T/T): 7.37). As seen with *UBA6*, the homozygous alternate individuals (T/T) have the weakest response to ER stress when taking control levels into account. The alternate allele (T) (allele frequency = 0.42) is also the ancestral allele. Our data suggest that natural genetic variation within a potential ERSEII binding site might alter the transcriptional regulation of *RABEP1* under ER stress conditions. This may impact protein homeostasis within the cells via endosomal trafficking and ER contact sites.

The top significant result with the alternate allele having higher expression levels under ER stress conditions, as opposed to the reference allele, is for a variant within a UPRE motif 2,670 bp upstream that impacts the gene *Phosphoserine Phosphatase Homolog Pseudogene 1* (*PSPHP1*) (*P*-adj = 2.2 × 10^−17^) (Fig. 5c). Under ER stress conditions, individuals who are homozygous for the alternate allele have a higher expression of *PSPHP1* (average TMLog2Intensity: homozygous reference (C/C): 10.50; heterozygous (C/T): 11.62; homozygous alternate (T/T): 12.48). While individuals who are homozygous alternate have an elevated expression level under ER stress conditions, those who are homozygous reference have a much higher fold change in response to ER stress (fold change: C/C = 3.3, C/T = 1.6, T/T = 1.4). This implies that while individuals who are T/T have a higher expression under ER stress conditions, individuals who have a C/C genotype have expression levels that are much more responsive to ER stress conditions. The alternate allele (T) (allele frequency = 0.29) is also the ancestral allele. *PSPHP1* is a pseudogene of *PSPH* that contains a region of exact homology. *PSPH* is a cytosolic protein that is involved in serine biosynthesis (Collet et al. 1997, 1999). While there is no published functional data for *PSPHP1*, multiple independent studies have confirmed a PSPHP1 transcript and have noted a strong correlation of expression of this pseudogene with cancer (Wallace et al. 2008; Martin et al. 2009; Wei et al. 2011; Allard et al. 2012). The link between the UPR and cancer has long been recognized (Yadav et al. 2014). We hypothesize that *PSPHP1* expression is regulated through a UPRE motif located upstream. This provides a potential role for *PSPHP1* in the UPR and the already noted association of PSPHP1 and cancer.

Further functional validation would be required to test whether the putative binding motif near the gene of interest is a true binding site of the relevant TF and how the SNP from our *cis*-eQTL analysis may impact binding efficacy and gene expression. Additionally, in each of these examples, the alternate allele that impacts gene expression is the ancestral allele. This could provide clues as to how these sites are evolving, but further investigation would be required to determine if this pattern holds true for other sites and how they impact expression. Our results are an important resource for identifying novel TFBSs and ER stress genes that may underlie the variable human ER stress response.

## Conclusion

By addressing the 4 questions outlined in the introduction, our study produced a pipeline for identifying potential TFBSs, and it produced lists of genes and variants that putatively cause variation in the ER stress response. We used publicly available DNA sequence and gene expression data sets to perform the first *cis*-eQTL analysis on human cell lines after the induction of ER stress. We also discovered genetic “hotspots” that could impact TF binding and gene expression, potentially contributing to tissue-specific disorders such as heart disease, type 2 diabetes, and prostate cancer (Fogarty et al. 2014; Huang et al. 2014; Reschen et al. 2015). These findings may help explain tissue-specific effects of the ER stress response, which is crucial for understanding many human diseases.

Our work provides a foundation for future research to investigate the role of TFBSs in the ER stress response and to identify new targets for drug discovery. By leveraging computational approaches with experimental data, we can further understand the genetic basis of the ER stress response and its implications for the development of treatments for ER stress-related diseases.

All data, scripts, and algorithms used in this study are publicly available, allowing our results to be reproduced and our pipeline applied to many other TFs in addition to the 3 well-known ones analyzed here. These applications will be facilitated by the public availability of our generated data and results. While we provided cutoffs for filtering and justification for each, further work should also evaluate the significance of varying these cutoffs for criteria such as consensus sequence, proximity to a gene, and conservation scores. We hope this work can be extended beyond the 3 TFs studied here and used as a guideline or resource for others who are interested in the computational identification of TFBSs in pathways other than the ER stress response.

## Data Availability

Code used for analysis can be found at Zenodo (https://doi.org/10.5281/zenodo.7958239) and GitHub (https://github.com/RogueRussell/Motif_Paper_Code). Supplemental material available at figshare (https://doi.org/10.25387/g3.23979093).
